# Development of Spatial Orientation in Two-to-Three-Year-Old Children in Relation to Lifestyle Factors

**DOI:** 10.3390/nu14163322

**Published:** 2022-08-13

**Authors:** Claudia van Dun, Ilaria Lisi, Janna van Diepen, Gabriele Gross, Gabriele Janzen, Esther Aarts

**Affiliations:** 1 Donders Institute for Brain, Cognition and Behaviour, Radboud University, 6525 EN Nijmegen, The Netherlands; 2 Medical and Scientific Affairs, Reckitt|Mead Johnson Nutrition Institute, 6545 CJ Nijmegen, The Netherlands; 3 Behavioural Science Institute, Radboud University, 6525 GD Nijmegen, The Netherlands

**Keywords:** lifestyle, cognition, development, toddlers, egocentric, allocentric, virtual reality, screen time, gestational weight gain

## Abstract

Various lifestyle factors, including diet, physical activity, and sleep, have been studied in the context of children’s health. However, how these lifestyle factors contribute to the development of cognitive abilities, including spatial cognition, remains vastly understudied. One landmark in spatial cognitive development occurs between 2.5 and 3 years of age. For spatial orientation at that age, children learn to use allocentric reference frames (using spatial relations between objects as the primary reference frame) in addition to, the already acquired, egocentric reference frames (using one’s own body as the primary reference frame). In the current virtual reality study in a sample of 30–36-month-old toddlers (*N* = 57), we first demonstrated a marginally significant developmental shift in spatial orientation. Specifically, task performance with allocentric performance increased relative to egocentric performance (*η_p_*^2^ = 0.06). Next, we explored a variety of lifestyle factors, including diet, in relation to task performance, to explain individual differences. Screen time and gestational weight gain of the mother were negatively associated with spatial task performance. The findings presented here can be used to guide future confirmatory studies about the role of lifestyle factors in the development of spatial cognition.

## 1. Introduction

In the past decades, there has been a dramatic rise of overweight and obesity amongst children worldwide [1]. Modifiable lifestyle factors, such as what children eat, and how much they sleep and exercise or spend time watching television, have an impact on children’s physical well-being, and this includes brain and cognitive development [2]. Research on the relative contribution of these factors to cognitive development is imperative for children to reach their full potential, which in turn is beneficial for society as a whole.

One brain structure that is especially prone to external influences, such as the aforementioned lifestyle factors, is the hippocampus [3]. A possible explanation for this sensitivity of the hippocampus is its susceptibility to neuroinflammation [3] and its neuroplasticity, which plays a crucial role in cognitive development [4]. The hippocampus is also the key brain hub for spatial cognition [5]. Spatial cognition can be defined as the knowledge and cognitive representation of the structure, entities, and relations of space [6]. It is a multifaceted construct, including, amongst other subdomains, spatial orientation and navigation.

Given the hippocampus’ sensitivity to external influences and its key role in spatial cognition, lifestyle factors may have a pronounced effect on spatial cognitive development. However, evidence for this hypothesis stems mainly from rodent studies. For example, one study reported that rodents consuming a high-fat diet for 8–10 weeks showed impairments in spatial navigation abilities later in life, compared with chow-fed littermates [7]. The high-fat diet also promoted an exaggerated inflammatory response to immune challenge, particularly in the hippocampus [7]. Importantly, these marked impairments were only visible in juvenile exposure to a high-fat diet, and not in adult exposure. This study highlighted the particular sensitivity of the hippocampus to diet-induced changes early in life that impact spatial cognition.

There is also evidence for an association between higher intakes of saturated fat and poorer hippocampal-dependent abilities in prepubescent children [8]. Another diet constituent that might play a role is dietary fiber, which is important for the gut microbiota [9], which in turn can affect inflammation [10]. Accounting for both dietary quality and quantity is important, given that malnutrition due to either suboptimal caloric intake or suboptimal nutrient levels, or both, has been related to impaired cognitive development [11]. Moreover, both underweight and overweight/obese children showed diminished cognitive development [11,12].

Other lifestyle factors affecting inflammatory processes are related to physical activity [13]. Emerging evidence indicates that physical activity is beneficial to cognitive development, even in early childhood (<5 years of age) [14]. A related lifestyle factor, contributing to sedentary behavior, is screen time. Excessive screen time was already found to be associated with reduced cognitive abilities in children below 36 months of age [15].

In addition to diet and physical activity, sleep is a modifiable lifestyle factor that may contribute to children’s cognitive functioning, due to its large impact on the immune system [16]. One study showed that sleep duration in infancy was associated with better executive functioning at 4 years of age [17]. Moreover, shorter sleep duration has been demonstrated to be related to lower cognitive performance in school-aged children, including visuo-spatial abilities (see [18] for a review).

Some lifestyle factors may already impact cognitive development before a child is born, or shortly thereafter. For example, gestational weight gain of the mother either below or above the recommended weight gain has been related to reduced cognitive performance in the offspring [19]. Moreover, longer breastfeeding duration is generally found to be associated with better cognitive abilities, including spatial skills, later in life [20].

Related to cognitive development, successful spatial orientation and navigation can be supported by adopting two fundamental reference frames to represent location [21]. In an egocentric reference frame, locations are represented in relation to the observer, whereas in an allocentric reference frame, locations are coded independently from the observer’s position and are instead coded based on inter-object relations [22]. From a developmental perspective, egocentric coding is acquired earlier than allocentric coding. Twelve-month-old infants can rudimentarily use egocentric orientation for coding object locations [23]. Egocentric orientation is mostly subserved by the precuneus, inferior and superior parietal cortices, and the frontal cortex [24]. Allocentric orientation is often considered a more advanced orientation strategy, and it is mostly prevalent in the later stages of development [25]. During development, younger toddlers have been shown to primarily use egocentric-related spatial cues for orientation, whereas older toddlers also incorporate allocentric information [25]. In particular, Bremner and colleagues report an improvement in the efficiency of spatial cue use for orientation between the ages of 24 and 36 months [26]. Preferential choice of the allocentric orientation strategy begins only during early school age [27]. Allocentric orientation is mostly subserved by the hippocampus [28], the region in which neurons with allocentric properties have been found, both in rats [29] and monkeys [30]. In humans, hippocampal maturity has been associated with increased allocentric memory abilities [31].

Given the presented evidence of a sensitive period between 24 and 36 months for the development of spatial cue use for orientation [26], the orientation abilities in a younger (30 months old) and an older (35 months old) group of toddlers were investigated using an on-screen VR paradigm [32]. Participants had to locate a target after a rotational transformation. Using visual spatial cues for maintaining orientation was more successful in 35- vs. 30-month-old children. Moreover, older children could use allocentric orientation to perform the task when needed, whereas younger children relied solely on egocentric coding [32]. Of note, individual differences in daily living skills (assessed through the Vineland-Screener questionnaire) predicted orientation success. Together, this study demonstrated that the developmental period between 30 and 35 months is highly sensitive for spatial cue use and that ability to use allocentric cues is present in older vs. younger children. Importantly, the results also highlight that, on top of chronological age, individual differences exist that contribute to determining successful use of visual cues for orientation.

We hypothesize that modifiable lifestyle factors could add to individual differences in spatial task performance in children, given the role of the hippocampus in spatial cognition and its sensitivity to neuroinflammation. However, most research in this area has focused on lifestyle factors in the context of children’s health, as opposed to children’s cognition [2]. The relation between lifestyle factors and the shift of egocentric to allocentric coding in toddlers has not been explored. Therefore, the aim of the current study was twofold. First, we aimed to confirm the development of egocentric and allocentric orientation abilities in a group of toddlers ranging from 30- to 36-months of age. We used an adjusted version of the on-screen VR task mentioned above to assess spatial abilities [32]. Differently from Van der Brink and Janzen’s study [32], we used age as a continuous rather than dichotomous variable, to obtain a more nuanced understanding of the role of age in spatial cognitive abilities, e.g., allowing us to investigate whether spatial cognition develops more linearly or stepwise within this age range. In line with previous findings, we hypothesized that performance would be better in trials that require egocentric orientation, compared with trials that require allocentric orientation, and we expected performance to generally increase with age. Additionally, we hypothesized that with age, allocentric orientation abilities increase relative to egocentric orientation abilities. Secondly, we aimed to explore various lifestyle factors, to explain individual differences in spatial task performance using variables derived from three parent questionnaires (Food Frequency Questionnaire, Vineland-Screener, and an additional lifestyle questionnaire). Lifestyle factors included dietary quality, caloric intake, saturated fat ratio, fiber intake, body mass index (BMI), sleep duration, exercise, screen time, gestational weight gain of the mother, and breastfeeding duration. This second aim was exploratory, and therefore no specific hypotheses regarding this part were formulated at the study outset.

## 2. Materials and Methods

### 2.1. Participants

A total of fifty-seven toddlers (31 girls, 26 boys), between 30 and 36 months of age (*M*_age_ = 33.05 *SD*_age_ = 1.87) completed the study. Parents were invited from the Baby and Child Research Center of the Radboud University Nijmegen in The Netherlands to participate in the study. Both over-the-phone and in-person screening were carried out to ensure participants met the inclusion criteria (born within 37 and 42 gestational weeks; no deafness, blindness, or other senso-motoric handicaps or reduced hand-eye coordination; no diabetes; no chronic inflammatory diseases (e.g., asthma, Crohn’s disease); no epilepsy; no brain surgery; no drastic changes in the diet in the past year; no daily usage of ibuprofen, aspirin, or other form of medication (except from homeopathy or vitamin supplements); no tooth extractions in the past month; no vaccinations in the past month; no antibiotics in the past three months). The education level of our sample (i.e., highest education obtained by either one of the parents) was 1.8 percent low, 10.9 percent middle, and 87.3 percent high. For context, the average education level in 2018 in the Netherlands was 29 percent low, 40 percent middle, and 30 percent high [33]. The sample was generally homogeneously highly educated, and therefore control for education level in our analyses could not be done. The consequences of this for the generalizability of the results will be elaborated on in the discussion. Participation in the study was voluntarily, and parents received 20 euros as reimbursement for their time. Written informed consent was obtained from the parents. The study was approved by the local ethical committee of Social Sciences of the Radboud University and the local ethical committee for human research of Arnhem and Nijmegen.

To answer our primary research question, an analysis of the interaction between a within-subject factor (Condition: congruent, incongruent) and a covariate (Age) was required. This design is similar to that of a regression analysis with one predictor (Age), using the difference scores (congruent–incongruent) as the outcome variable; a design that can be used for a power analysis in G*Power. We based the effect size on the study by Van den Brink and Janzen [32], which used the same task, in the same age group. Thus, the a priori power analysis for a regression analysis with one predictor, an effect size of 0.23, an alpha of 0.05, and a desired power of 80 yielded a minimum sample size of 37. A power calculation with the same settings but using three predictors (Condition, Age, and Condition × Age) yielded a minimum sample size of 52.

### 2.2. Materials and Procedure

#### 2.2.1. BMI

Body mass index (BMI) is an internationally used measure that can be used from two years of age to assess healthy weight for height, through the following formula:

BMI = weight (kg)/height^2^ (m)

Alternatively, BMI z-scores can be calculated accounting for sex and age. We calculated BMI z-scores using the calculator from the World Health’s Organization. The correlation between the BMI z-scores and the normal BMI scores calculated using the formula above was *r* = 0.99, *p* < 0.001. Given the high correlation between both measures, we decided to include the more frequently used normal BMI scores in the exploratory analysis.

The experimenter measured the height and weight of the participating child and the accompanying parent at the research facility after the testing session. In addition, parents self-reported the height and weight of the participating child, of both parents and of siblings (if applicable) in the additional questionnaire (described below). For the exploratory analysis, we included the BMI of the child based on the measurement obtained during the test session.

For the current maternal BMI, we calculated the correlation between the data obtained by the experimenter and the self-reported data from the questionnaire based on the available data (*n* = 31, for mothers who accompanied their child to the test session), and the correlation was *r* = 0.99 (*p* < 0.001). For the mothers that did not accompany their child to the test session, half of the mothers (*n* = 13) self-reported a BMI in the questionnaire and the other half (*n* = 13) had missing data for this variable.

#### 2.2.2. Questionnaires

##### FFQ

To assess dietary quality, parents were asked to complete a Food Frequency Questionnaire (FFQ) in an online portal from their home computer. The FFQ for adults [34] was adapted by The University of Wageningen to meet specific dietary requirements for toddlers and extended to allow quantitative analyses about total energy consumption and macronutrients (see below). The FFQ assesses dietary intake, by querying the frequency of consumption of listed food items over a specified period of time (here, over the past month). Completion of the FFQ lasted approximately 25 min.

Data from the FFQ was pre-processed by the University of Wageningen, yielding index scores reflecting adherence to the Dutch Healthy Diet (DHD) guidelines [35] on eleven subcomponents (vegetables, fruit, whole wheat products, legumes, nuts, dairy, fish, fat and oils, highly processed meat (mirrored), sugary beverages (mirrored), and unhealthy food choices (mirrored)) on which a maximum of 10 points per subcomponent could be obtained. Thus, the total DHD-index scores used in the exploratory analysis, presented below could range between 0 and 110, with a higher score meaning better adherence to the Dutch guidelines for a healthy diet [35]. This measure was used to assess dietary quality. In addition to the DHD-index scores, total energy consumption, macronutrient composition of the diet, and fiber intake were calculated.

For the exploratory analysis, we used the total DHD-index scores, average energy consumption per day in kilo calories (kcals), the percentage of total kcals derived from average daily saturated fat intake, and average daily fiber intake in grams.

##### Vineland Screener

The second parent-completed questionnaire via a home computer was the Dutch Vineland Screener 0-6 (Vineland-S) [36], which was also applied in the previous study to explore individual differences in task performance. The Vineland-S is a behavioral questionnaire used to assess the adaptive developmental age of children under 6 years of age. Parents score their child’s everyday behavior across 72 items divided across four domains: communication skills, social skills, daily living skills, and motor skills [36]. The Vineland-S takes approximately 15 min to complete. Total scores are provided as age-equivalent scores for each domain, and a composite age-equivalent score per child. To prevent issues with multicollinearity, we did not include the composite scores in the exploratory regression analyses, and instead focused on the four separate domains.

##### Additional Lifestyle Questionnaire

The third parent-completed questionnaire via a home computer was a lifestyle questionnaire. The questionnaire included questions about the child’s habitual sleep and exercise, questions about the pregnancy period, feeding practices, general health and mouth hygiene of the child, and demographics of the family. In addition, parents were asked to measure the height, weight, and waist circumference of themselves and any siblings (if applicable). The duration of completing this questionnaire was approximately 15 min.

The demographic section included questions about the highest education level of either of the parents. Scores ranged from 1 to 5, indicating primary school level to university level, respectively. We used the maximum of these two scores to calculate maximum education level of the parents as a proxy of socioeconomic status. The education level of the parents was too homogeneous and high to be able to control for socioeconomic status in our analyses (see Participants section above).

Additionally, we derived a set of variables of interest from this questionnaire, to be included in the exploratory analysis. For *Sleep*, the average actual hours of sleep during the night and the average hours of sleep during the day were added. For *Exercise*, the average hours of outside play and sports per week were included. For *Screen time*, the total hours per week that a child watched television and used a computer, laptop, tablet, or smartphone were captured. For *Gestational weight gain* the total weight gain during pregnancy in kilograms was used. We did not ask pre-pregnancy BMI, although we did assess current maternal BMI. However, it should be noted that the latter may not be a good representation of normal BMI for the mothers, since many of them were currently pregnant, breastfeeding, or had just given birth. For *Breastfeeding duration*, the number of months the child was breastfed was used (0 if the child was never breastfed), irrespective of whether a child also received formula.

#### 2.2.3. Spatial Cognition Assessment

##### Stimuli

Spatial cognition was assessed with a virtual reality game using the same stimuli as employed by van den Brink and Janzen [32]. However, no trials containing landmarks were included, as they were shown in the original experiment to be distracting to participants. Stimuli consisted of 8 movies featuring a purple animated bird (target) appearing to the front of the screen, turning around and flying to hide in one of two identical trees. The trees were situated, at different distances, into one of four different types of 3D environments. All stimuli were designed using Blender, an open-source animation suite (www.blender.org). Stimuli were presented using a Dell computer running Presentation software (Neurobehavioral Systems, Inc., Berkeley, CA, USA).

**Figure 1:**
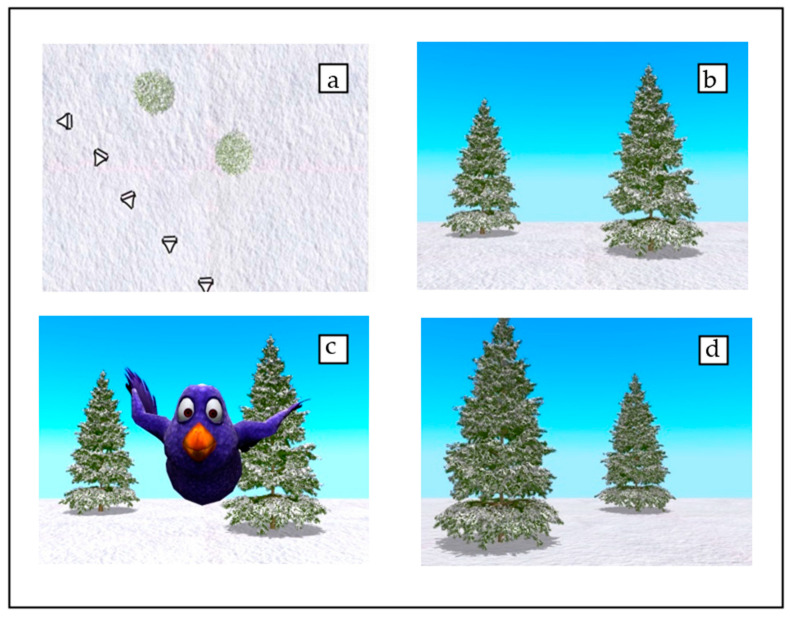
Camera path for an example trial. Panel (**a**) shows the top view of the camera path, indicating camera angle and position at five points. Panels (**b**,**d**) show the initial and final position of the camera, resulting in a 90° turn to the left of the center of the visual scene. After the initial image (**b**), Pico the bird appears (**c**) and hides in one of the two trees. Next, the camera shifts, resulting in the final image (**d**). Finally, children are asked to indicate in which tree Pico is hidden by pressing the correct tree on the touchscreen. (Image and caption adapted from [32]).

Once the bird had hidden itself in the tree, a camera shift mimicked a self-motion path (duration: 4 s). This led to a perspective change of 90°, to the left or the right of the center of the visual scene (see Figure 1). During the camera rotation, all objects in the environment temporarily disappeared from sight, preventing toddlers from keeping their eyes fixed on the target’s hiding position. Position of the hiding tree (left/right and front/back) and turn (90° left/right) were fully counterbalanced. For further details regarding the camera movements please refer to the stimuli section in Van den Brink and Janzen [32].

**Figure 2:**
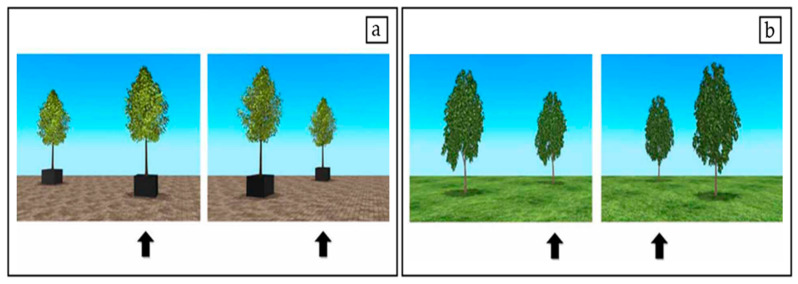
Experimental conditions of the spatial cognition task. The stills from the experimental movie depict the two trial types included in the present study. In side-congruent trials (SCon, panel **a**) the initial and final target position are on the same side of the participant’s body. In side-incongruent trials (SInc, panel **b**), the initial and final target position are on the opposite side of the participant’s body. Reliance on egocentric coding to respond on SInc trials results in below chance performance. (Image and caption adapted from [32]).

In total, the experiment contained 32 trials. In half of the trials, the tree in which the bird was hidden was on the same side of the participant’s body before and after the camera rotation; these were labelled side congruent trials (SCon, Figure 2a). Correct performance on SCon trials could be achieved either by egocentric or allocentric spatial coding. In the other half of the trials, labelled side incongruent trials (SInc, Figure 2b), the final hiding position was on the opposite side of the participants’ body compared to the hiding position before the camera turn. Correct performance on the SInc trials could only be achieved by allocentric spatial coding. Reliance on egocentric coding to respond on SInc trials would result in below-chance performance on these trials.

##### Procedure

All procedures were carried out in accordance with the regulations of the Baby Research Center of the Radboud University (Nijmegen, The Netherlands). Parental informed consent was collected, and exclusion criteria checked prior to starting the experiment. Upon visit to the research center, toddlers were seated on a highchair, or on their parents’ lap, depending on the child’s preference, in front of a touch screen monitor (Hewlett-Packard 23-inch LCD). A web cam (LG) was fixed on top of the monitor to record the child’s face and eye movements for the purpose of excluding non-attended trials (on average 10.5% of all trials). Throughout the experiment, the experimenter was seated on the left side of the child.

Participants were informed that the game they were about to play involved a bird named Pico, which they watched fly and hide behind one of the two trees present on the screen. Upon hiding, a camera shift led to a change in perspective of 90° either to the left or the right of the center of the visual scene (see Stimuli). Thereafter the child had to indicate, by touching the monitor, which tree the bird was hidden in. Cartesian coordinates of the touch were recorded, to store information about correct and incorrect responses.

Following the touch response, Pico would fly out of the correct tree providing the child with feedback on the correct hiding position. If the participant had chosen the correct tree, the bird chirped while flying toward the camera; otherwise, the bird made no sound. The first trial was always an example trial completed by the experimenter, to clarify the procedure for participants. In between each trial, as an incentive, children were allowed to stamp an offline paper sheet. After every 4 trials, children got to choose a sticker and place it on the sheet. In total, participants could complete up to 32 trials (*M* = 17.38 completed trials, *SD* = 4.41).

At completion of the experiment, the height and weight of children and the present parent were measured to later calculate BMI. Afterwards, several biological samples were collected from the child (buccal swab, saliva for proteomics and volatiles, and a stool kit for collection at home). Results regarding the biological data falls beyond the scope of this research paper and will be discussed elsewhere. Finally, parents were explained the procedure to complete the questionnaires at home and received compensation for their child’s participation in the study. The overall experimental procedure lasted for around one hour.

The setup for this experiment originally also involved functional near-infrared spectography (fNIRS). Measurements of the first 5 children therefore also included neuroimaging data. However, due to technical issues during the measurements and restrictions related to COVID-19, fNIRS measurements were terminated, and the experiment continued behaviorally. Data of the subset of children that received fNIRS did not differ substantially from the rest of the dataset and was therefore included in the dataset used for behavioral analyses.

### 2.3. Data Analyses

For our first aim, we performed a repeated-measures ANCOVA, to test for a developmental shift in performance, as found by Van den Brink and Janzen (2013), using age as a continuous variable. For this analysis, we used *Congruency* (side congruent, side incongruent; qualitative) as a within-subject factor and standardized *Age* (as a fraction of a year; quantitative) as a moderator or covariate of interest. *Percentage correct* was used as the dependent variable. 

For our second aim, we explored whether lifestyle factors were related to individual differences in task performance. We performed a regression analysis using *Mean percentage correct* on both trial types as the outcome variable, and with the following variables as predictors: *Age*, *Sex*, the standard scores on the four domains of the Vineland-S (*Communication* skills, *Daily living* skills, *Socialization* skills, and *Motor* skills), *Sleep*, *Exercise*, *Screen time*, *Dutch Healthy Diet (DHD) total score*, *Caloric intake*, *Saturated fat ratio*, *Fiber intake*, *Child BMI*, *Breastfeeding time*, and *Gestational weight gain* of the mother. Sex was added as a dummy variable and all other predictors were quantitative.

Given the exploratory nature of this analysis, *p*-values were of less importance here and were added for completeness. Instead, standardized regression weights (*β*) are often used to determine relative predictor importance. However, *β*-weights are highly sensitive to multicollinearity [37]. Due to this limitation, some authors have argued that structure coefficients should be added to results, to allow for correct interpretation [37]. Structure coefficients are the Pearson’s correlations between the predicted criterion score (Yhat, *Ŷ*) and each of the predictors. In the current study, there were no issues with multicollinearity (all tolerances > 0.35 and all VIFs < 2.83). Nonetheless, to allow for better interpretation of the results, we also included the structure coefficients for each of the predictors. Due to the limited sample size for the number of predictors used in the model, we will focus on those predictors that are of relevance based on both the *β*-weights and the structure coefficients.

Cohen’s conventions were used to evaluate the magnitude of the effect sizes. Thus, a correlation of 0.1 was considered small, 0.3 medium, and 0.5 large, and a partial eta-squared of 0.01 was considered small, 0.06 medium, and 0.14 large [38]. Differences of *p* < 0.05 were considered significant and *p* = [0.05–0.1] marginally significant.

We used R Studio and R version 3.5.1 with the following packages to process and visualize the data: Tidyverse version 1.2.1, Ggplot2 version2.2.1, Foreign version 0.8–71, and Haven version 1.1.2. We used IBM SPSS Statistics version 23 for the statistical analyses.

## 3. Results

### 3.1. Spatial Cognition Task Performance and Age

First, we confirmed that our data met the assumptions of normality, homogeneity, and linearity. The first aim of this study was to investigate the development of allocentric orientation abilities in toddlers between 30 and 36 months of age. We expected allocentric performance (reflected by performance on the incongruent trials) to increase with age relative to egocentric performance (reflected by performance on the congruent trials), which would be reflected in a significant *Congruency* × *Age* interaction effect. The results of the repeated-measures ANCOVA showed a significant main effect of *Congruency* (multivariate *F*(1,56) = 20.74, *p* < 0.001). This effect was large in size (multivariate *η_p_*^2^ = 0.27). Estimated marginal means indicated that the percentage correct was higher for the side congruent trials (*M* = 74.61%) than for the side incongruent trials (*M* = 56.02%). This means that egocentric performance was better developed in the toddlers than allocentric performance, as expected.

**Figure 3:**
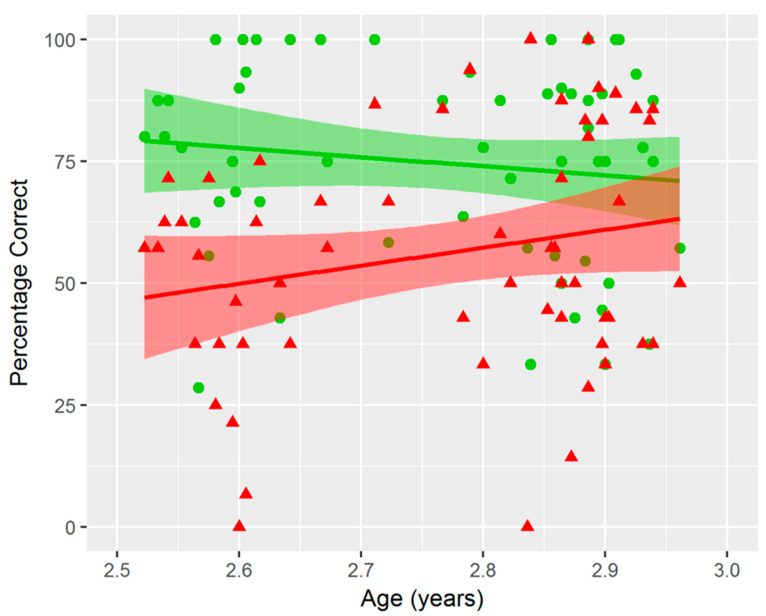
Main analysis results per subject for *Congruency* by *Age* on *Percentage Correct*. SCon means are represented in green circles, SIncon means in red triangles. Lines represent the regression lines, shaded area represents standard errors (SCon in green, SIncon in red).

In addition, we observed a marginally significant *Congruency* × *Age* interaction (multivariate *F*(1,56) = 3.76, *p* = 0.058), reflecting a developmental change across congruency conditions and , thus, consistent with the earlier findings of Van den Brink and Janzen [32]. This effect was medium in size (multivariate *η_p_*^2^ = 0.06). Parameter estimates indicated that the b-weight for side congruent trials was negative (*b* = −2.71), whereas the b-weight for the side incongruent trials was positive (*b* = 5.28), although both b-weights were not significantly different from zero (*p* = 0.33, and *p* = 0.11, respectively). A visual overview of these results is displayed in Figure 3.

The main effect of *Age* was not significant (*F*(1,56) = 94.06, *p* = 0.55).

### 3.2. Relation between Spatial Cognition Task Performance and Lifestyle Factors

**Figure 4:**
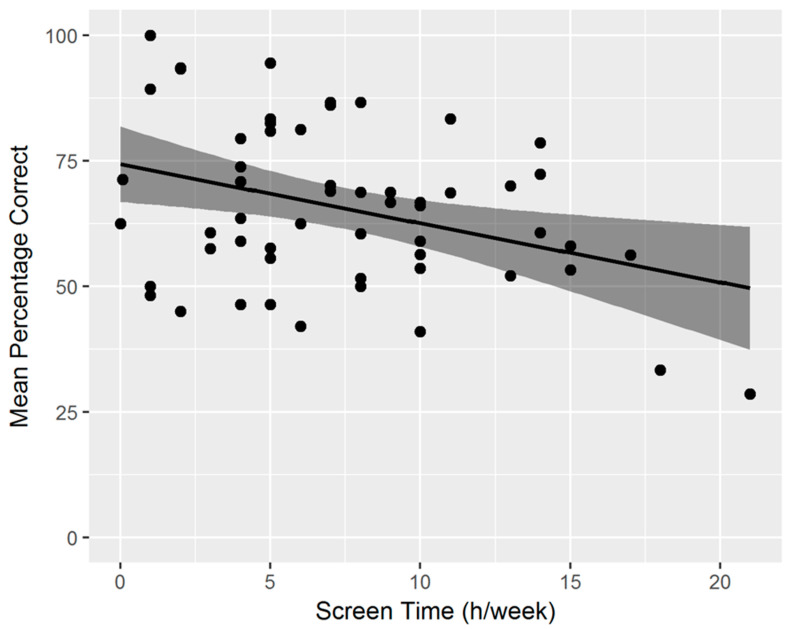
The relation between screen time and task performance (mean percentage correct).

**Figure 5:**
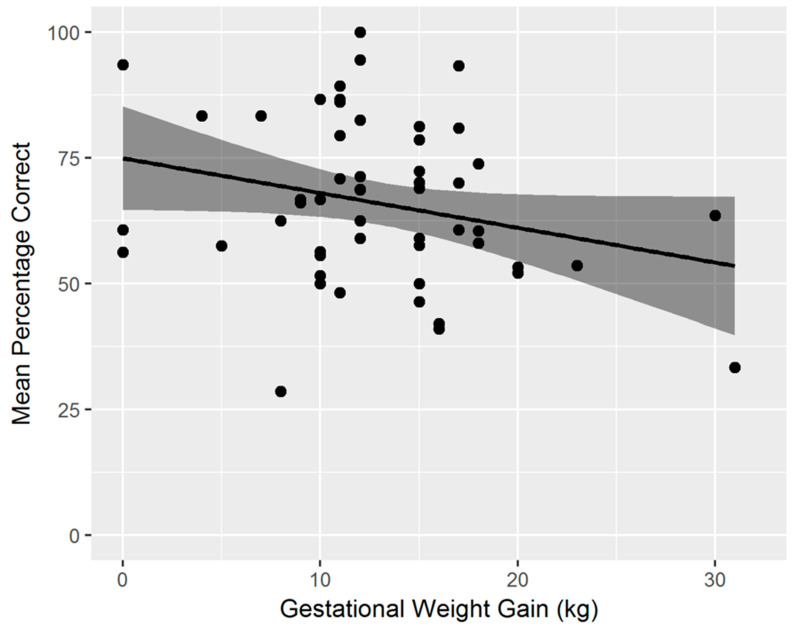
The relation between gestational weight gain and task performance (mean percentage correct).

After confirming similar developmental effects with age, as described earlier [32], we wanted to assess what factors, in addition to age, contributed to performance on this task, by exploring the dataset in a regression analysis. See Table 1, for the description of all variables and their correlations. The combination of lifestyle factors explained a good proportion of the variability in spatial cognition task performance (*R*^2^ = 0.45), resulting in a significant model (*F*(16,38) = 1.97, *p* = 0.04). An overview of all corresponding *β*-weights, *p*-values, and structure coefficients (*r*) can be found in Table 2. Based on a combined evaluation of *β*-weight and structure coefficient, the most important predictor for overall task performance (% correct) was *Screen time* (*β* = −0.69, *p* <.001; *r* = −0.58, *p* < 0.001), with more screen time being associated with decreased task performance (see Figure 4). The second most important predictor was *Gestational weight gain* (*β* = −0.31, *p* = 0.03; −0.38, *p* = 0.005), showing that a lower weight gain of the mother during pregnancy was associated with increased task performance of their child (see Figure 5). For further interpretation of the results, we correlated gestational weight gain with current maternal BMI, as far as data was available (*n* = 44). The correlation was *r* = −0.27 and marginally significant (*p* = 0.07). This effect was of medium size and indicated that mothers that generally gained more weight during pregnancy had a lower current BMI. All other predictors were of relevance on the structure coefficients only and not on the *β*-weights, or not relevant on either, and we will therefore not further discuss these, given their limited statistical power.

## 4. Discussion

The aim of the current study was twofold: (1) to investigate the spatial orientation abilities in toddlers as a developmental milestone with increasing age, and (2) to explore a variety of lifestyle factors that could possibly explain individual differences in spatial abilities.

To investigate the development of egocentric and allocentric orientation abilities in toddlers, we used an adjusted version of an on-screen VR task to assess spatial abilities [32]. Differently from Van der Brink and Janzen’s [32] study, we used age as a continuous rather than dichotomous variable, to allow a more nuanced understanding of the role of age in spatial cognitive abilities in this age range (30–36 months of age). We hypothesized that performance on the congruent trials would generally be better than on the incongruent trials. Additionally, we expected performance on the incongruent trials, for which allocentric processing is required, to increase with age relative to performance on congruent trials, for which egocentric processing alone would be sufficient. Indeed, we found that performance on the congruent trials was generally superior to that on incongruent trials, confirming our first hypothesis. The interaction between condition and age was marginally significant, and the effect size was medium, providing some evidence in the direction of our second hypothesis. These findings are generally in line with the results of the study by Van den Brink and Janzen [32]. However, contrary to the findings in that study, we did not find a significant main effect of age, despite having a large enough sample according to the a priori power calculation. One possible explanation for this difference is that we included children in the age range of 30–36 months, as opposed to including only the extreme age groups (30 months versus 35 months). The latter approach generally has more power [39] and is perhaps more successful in finding an effect when the shift is gradual or when a sudden shift happens more towards the beginning or the end of the age range. Future studies should elucidate whether this development is more gradual or at a sudden moment in time between 30 and 35 months of age. Cognitive neuroimaging might be able to link these developmental steps to stages in brain maturation, such as that of the hippocampus [28].

For exploring lifestyle factors in relation to spatial task performance, we used an exploratory regression analysis using a variety of demographic (Age, Sex) and lifestyle factors (Sleep, Exercise, Screen time, Dutch Healthy Diet (DHD) total score, Caloric intake, Saturated fat ratio, Fiber intake, Child BMI, Breastfeeding time, and Gestational weight gain of the mother) as well as the standard scores on the four domains of the Vineland-S (Communication skills, Daily living skills, Socialization skills, and Motor skills) as predictors. Of the factors explored here, only Screen time and Gestational weight gain were negatively associated with spatial task performance in toddlers. The null results of the remaining factors included in the sample will not be further discussed, because the lack of an effect could also have been due to insufficient power, given the limited sample size. Future studies using larger samples could further explore these factors and their interactions, to obtain a better insight into the more subtle relations between lifestyle factors and spatial task performance in toddlers.

In most previous studies, clear negative associations between screen time and cognitive development in young children only became apparent for extremely high exposure (i.e., more than 7 h per day in some studies) [40]. The average screen time use in our study was relatively low (*M* = 7.51 h/week, *SD* = 1.07), and within the general guidelines for children this age (i.e., <2 h/day). Nevertheless, we still observed a negative correlation with spatial task performance. Screen time in our study also included playing games on a laptop or tablet. One might expect this gaming experience to be beneficial for playing a spatial cognition game on a touch screen computer, but the pattern observed here suggests the opposite. The negative correlation found in the current study could possibly be explained by a negative association between screen time and cognitive stimulation in the home environment. Indeed, one study in 24–36 month old toddlers found such a negative association for television viewing [41]. Screen time could have come at the expense of real-life play and associated spatial experiences, resulting in a negative association with spatial cognition. However, we did not assess real-life play duration in our study. Additionally, we only focused on screen exposure duration, and we did not specifically assess the type of content toddlers engaged with. Some studies have found that the type of media content (i.e., educational or non-educational; violent or non-violent) determines the impact on cognitive abilities [42]. Therefore, future studies could also include questions on the type of content, to further unravel this association.

The current findings with respect to gestational weight gain are not exactly in line with a longitudinal study using a large sample (*N* > 5000 children), which found a small positive association between gestational weight gain and offspring cognitive development [19]. An older study found that general cognitive ability (measured with the Raven Coloured Progressive Matrices) was higher for children of which the mother gained more than 2.27 kg (5lb) and less than 13.15 kg (29lb) [43], which suggests an inverted U-shaped relation between gestational weight gain and cognitive ability of the offspring. In our modest sample, gestational weight gain was relatively high (*M* = 12.98 kg, *SD* = 5.88), with very few (*n* = 3) mothers falling below the lower threshold, which could explain why we found a negative linear association, in line with these older findings. A more recent study using a large sample (*N* = 31,968), indeed, found an inverted U-shaped association [44]. However, comparing these results to those from a different model that accounted for sibling pairs in their data revealed that almost all observed associations could be explained by familial factors. Our sample size (*N* = 57) and composition did not allow us to control for possible confounders, other than those factors already included in the regression analysis, and should therefore be interpreted with caution. We encourage future confirmatory studies using larger samples to investigate the association observed here, including possible confounders such as socio-economic status, and using analyses that account for non-normally distributed data, such as generalized estimating equations (GEEs). Such studies are warranted, since the limited amount of studies to date on gestational weight gain and cognitive abilities of the offspring have led to inconclusive results (see [45] for a recent review and meta-analysis), and studies investigating spatial cognition specifically are currently lacking. One mechanism that could potentially explain the association between gestational weight gain and cognition of the offspring are inflammatory processes. Indeed, excessive gestational weight gain was found to be associated with higher concentrations of inflammatory markers in the mothers [46]. Inflammation during pregnancy may impact inflammatory profiles in the offspring, but evidence for this hypothesis mainly stems from rodent studies and more research in humans, investigating the causal mechanisms, is warranted [47]. Given the key role of the hippocampus in spatial cognition, and the sensitivity of this brain region to external influences and inflammation [3], spatial cognition could be a very suitable candidate to further investigate the consequences of heightened inflammatory factors during pregnancy on the cognitive abilities of the offspring.

The two lifestyle factors found to be (negatively) associated with spatial cognitive task performance in toddlers in the current study—screen time and gestational weight gain of the mother—are both modifiable. Although we cannot claim causality based on our correlational analyses in this observational study, future studies that use lifestyle intervention designs might show an impact on children’s cognitive abilities. By increasing awareness through education of (future) parents, the risk of negative consequences could be reduced. Helping children reach their full potential is particularly important when it comes to spatial cognitive abilities, because spatial orientation allows them to locate what they need [32]. Moreover, spatial abilities are linked to quantitative reasoning skills. Indeed, longitudinal studies have shown that increased spatial abilities are associated with proficiency in mathematics and science [48]. These skills are highly relevant for today’s society, where numeracy, the ability to analyze and interpret data, and critically assess complex problems are increasingly requested abilities.

One important limitation, particularly for the exploratory regression analysis with the lifestyle factors, is the limited sample size and homogenous study population. Results should therefore be validated in larger, more heterogenous cohorts. The vast majority (87 percent) of the parents of the toddlers in the current study sample were highly educated, which could be interpreted as a proxy of socioeconomic status. A more heterogeneous sample would increase the generalizability of the findings, especially since characteristics of the home environment have the potential to affect cognitive development [49]. As this observational study does not allow any causal inference, intervention studies in a randomized controlled design would allow for conclusions regarding the causal role of screen time or gestational weight gain in affecting spatial cognition in toddlers.

## 5. Conclusions

Using an existing touch-screen task for spatial cognition, we confirmed that egocentric orientation abilities are better developed in toddlers than allocentric abilities. We also found that allocentric versus egocentric performance tended to increase with increasing age between 30 and 36 months (medium effect size), in line with previous findings comparing groups of toddlers aged 30 versus 35 months. Regarding the role of various lifestyle factors in explaining individual variability in spatial cognition, a negative correlation for both screen time and gestational weight gain with performance on the spatial cognition task was found. These results need to be confirmed in larger studies, preferably using intervention designs, to claim causality.

## Figures and Tables

**Table 1:** Means, standard deviations, and Pearson’s correlations of the variables included in the exploratory regression analysis to investigate individual differences in task performance.

Predictor	*M*	*SD*	1	2	3	4	5	6	7	8	9	10	11	12	13	14	15
1. Age	2.77	0.12															
2. Sex	0.47	0.50	0.31 *														
3. Communication skills	41.44	6.15	0.32 *	0.22													
4. Social skills	41.91	9.15	0.27 *	−0.05	0.45 **												
5. Daily living skills	36.15	8.57	0.12	−0.24	0.42 **	0.55 **											
6. Motor skills	41.02	6.13	0.09	0.12	0.45 **	0.56 **	0.48 **										
7. Sleep	12.21	1.05	−0.05	−0.04	−0.30	−0.29 *	−0.26	−0.05									
8. Exercise	8.91	5.55	0.07	0.18	−0.01	0.02	−0.03	0.12	−0.05								
9. Screen time (h/week)	7.51	4.82	0.24	0.006	0.03	0.32 *	−0.15	0.22	−0.01	0.40 **							
10. Dutch Healthy Diet score	64.80	14.12	−0.02	0.09	−0.01	0.01	0.14	−0.01	−0.01	−0.21	−0.38 **						
11. Caloric intake	1111.50	287.03	0.25	0.35 **	0.03	0.08	−0.14	−0.008	0.03	0.19	0.11	0.13					
12. Saturated fat ratio	12.12	2.44	0.12	0.09	0.004	−0.09	−0.23	−0.12	0.24	−0.10	−0.07	−0.18	0.16				
13. Fiber intake	14.77	3.86	0.13	0.13	0.04	0.07	0.04	−0.007	0.02	0.02	−0.13	0.44 **	0.63 **	−0.10			
14. Child BMI	16.34	1.39	−0.04	0.005	−0.001	−0.20	−0.20	−0.13	0.16	−0.02	−0.23	−0.02	−0.18	0.23	0.02		
15. Breastfeeding time	8.65	8.71	−0.21	−0.38	−0.02	−0.05	0.13	−0.10	−0.02	−0.19	−0.25	0.19	−0.32 *	−0.22	−0.04	0.11	
16. Gestational weight gain	12.95	5.88	−0.01	0.12	−0.24	0.02	−0.03	0.08	−0.06	0.17	0.07	0.09	0.26	−0.11	0.18	−0.21	−0.15

*Note*. *M* and *SD* are used to represent mean and standard deviation, respectively. * indicates *p* < 0.05 and ** indicates *p* < 0.01 significance of correlation between the predictors (the numbers in the first row are explained in the first column). The highest correlation was *r* = 0.63 (between predictors 13 and 11).

**Table 2:** Significance of the lifestyle factors in the exploratory regression analysis predicting performance in the spatial cognition task (i.e., Mean percentage correct as the dependent variable).

Predictor	*β*	*p*	*r*	*p*
Age	0.05	0.75	0.05	0.72
Sex	−0.26	0.13	0.003	0.98
Communication skills	0.22	0.21	0.42	0.001 **
Social skills	0.18	0.35	0.15	0.28
Daily living skills	−0.21	0.31	0.28	0.04 *
Motor skills	0.12	0.49	0.11	0.41
Sleep	0.01	0.93	−0.07	0.59
Exercise	0.22	0.14	−0.08	0.58
Screen time	−0.69	<0.001 ***	−0.58	<0.001 ***
Dutch Healthy Diet total score	−0.006	0.97	0.21	0.12
Caloric intake	0.30	0.14	0.33	0.02*
Saturated fat ratio	0.009	0.95	0.16	0.25
Fiber intake	−0.02	0.92	0.29	0.03 *
Child BMI	−0.17	0.24	−0.04	0.77
Breastfeeding time	−0.10	0.48	−0.01	0.94
Gestational weight gain	−0.31	0.03 *	−0.38	0.005 **

Note. * indicates *p* < 0.05. ** indicates *p* < 0.01. *** indicates *p* < 0.001. *β* = standardized Beta-weight, *r* = structure coefficient.

## Data Availability

The data presented in this study are available on request from the corresponding author. The data are not publicly available, because no written consent for public data sharing was obtained from the parents of the participating children.
